# Supplementary material for: The genome sequence of the cephid sawfly, Cephus spinipes (Panzer, 1800)
Source: Wellcome Open Res. 2024 Sep 26;9:557. [Version 1] doi: 10.12688/wellcomeopenres.23068.1 (PMC11494279; doi:10.12688/wellcomeopenres.23068.1)
Supplement: Peer review history

# Reviewer report: Reviewer response for version 1

**DOI:** 10.21956/wellcomeopenres.25404.r103131

**Published:** 2024-10-21

**Authors:** Manee M Manee

**Affiliations:** 1 King Abdulaziz City for Science and Technology, Riyadh, Saudi Arabia

This study reports the first complete genome assembly of *Cephus spinipes*, a cephid sawfly. The assembly achieved high continuity and completeness (96.5% BUSCOs) and used a combination of long-read PacBio sequencing and Hi-C data. Additionally, the mitochondrial genome was assembled. This genomic data has the potential to impact research in insect genomics and evolutionary biology by offering new insights into sawfly genetics. I am happy with the current form of this manuscript.

Are sufficient details of methods and materials provided to allow replication by others?

Yes

Is the rationale for creating the dataset(s) clearly described?

Yes

Are the datasets clearly presented in a useable and accessible format?

Yes

Are the protocols appropriate and is the work technically sound?

Yes

Reviewer Expertise:

bioinformatics, genomics, and evolutionary biology

I confirm that I have read this submission and believe that I have an appropriate level of expertise to confirm that it is of an acceptable scientific standard.

# Reviewer report: Reviewer response for version 1

**DOI:** 10.21956/wellcomeopenres.25404.r103135

**Published:** 2024-10-19

**Authors:** Sophie Tandonnet, Rafath Chowdhury

**Affiliations:** 1 Universitat de Barcelona, Barcelona, Catalonia, Spain

In this genome note, the authors report the chromosomal-scale assembly of the genome of *Cephus spinipes* (238.6 Mb), the first complete Cephidae genome. The assembly is highly continuous and complete (96.5% complete BUSCOs) and originates from only two male individuals (specimen iyCepSpin1 for Hi-C and specimen iyCepSpin2 for PacBio long read sequencing). 

 The assembly is based on Pacbio long reads and scaffolded into pseudo-chromosomal molecules using Hi-C data. The assembly does not yet have a corresponding annotation, but the study did produce an RNA-seq dataset from the abdomen of a third individual (specimen iyCepSpin7).

 All in all this note reports a new, high quality genome assembly of a species not yet sequenced. 

 Below we make a few comments and suggestions for improvement:

- Add the information of the genome annotation as soon as it becomes available. Also why use only the abdomen for generating RNA-seq data? The more tissue types, the better the annotation can be.
- About the MT genome: did the authors check for duplications? 
- The authors mention: "The assembly is haploid, as this is a male sample"

This requires more explanations about the biology, genetics and sex determination of *C. spinipes* in the introduction. 

- The authors mention that "The order and orientation of contigs within the centromere-associated regions of chromosomes 1 to 8 is uncertain".

 Is there any hypothesis why this has happened?

 Visually (on figure 5), we could not see that chromosome 9 and 10 were much different from the others. How did the authors define the regions of uncertain order/orientation?

 The authors found *Wolbachia* as a cobiont and assembled its genome. This is quite nice and this information could be described in the report.

Are sufficient details of methods and materials provided to allow replication by others?

Yes

Is the rationale for creating the dataset(s) clearly described?

Yes

Are the datasets clearly presented in a useable and accessible format?

Yes

Are the protocols appropriate and is the work technically sound?

Yes

Reviewer Expertise:

Evolutionary biology, genomics, developmental biology

We confirm that we have read this submission and believe that we have an appropriate level of expertise to confirm that it is of an acceptable scientific standard.
